# The Relationship Between Gestational Diabetes and Postpartum Depression: A Systematic Review

**DOI:** 10.7759/cureus.64219

**Published:** 2024-07-10

**Authors:** Saeed Abdullah Saeed Alqahtani, Faris A Alasmre, Hind A Alasmre, Lujain A Alasmre, ‏Yousef M Mohammed, Norah Aljuaid, Faris Ali Rajeh Alzahrani, Saeed Jumaan Hamed Alghamdi, Yousef Mohammed Matar Alzahrani, Sobhia N Abanmi

**Affiliations:** 1 Department of Obstetrics and Gynecology, Abha Maternity and Children Hospital, Abha, SAU; 2 College of Medicine and Surgery, King Khalid University, Abha, SAU; 3 College of Medicine, King Khalid University, Abha, SAU; 4 College of Medicine and Surgery, Umm Al-Qura University, Makkah, SAU; 5 Department of Medicine, Al Baha University, Al Baha, SAU; 6 Department of General Practice, Bani Hasan Primary Health Care Center, Al Baha, SAU; 7 Department of General Practice, King Fahad Hospital, Al Baha, SAU; 8 College of Medicine, Majmaah University, Riyadh, SAU

**Keywords:** gestational diabetes, gestational diabetes mellitus, surgical outcomes, breast cancer, breast density

## Abstract

This study aims to examine the relationship between gestational diabetes mellitus (GDM) and the likelihood of postpartum depression (PPD) symptoms. PubMed, Scopus, Web of Science, ScienceDirect, and the Wiley Online Library were systematically searched for relevant literature. Our results included eight studies with a total of 4,209 women diagnosed with GDM and/or PPD. The prevalence of PPD in women diagnosed with GDM ranged from 6.5% to 48.4%. The included studies demonstrated that PPD was more likely to strike women with GDM. One study reported that the most severe type of GDM is more likely to occur in those with a history of depression. Perinatal depression during pregnancy can be strongly predicted by age, BMI, and a personal history of depression. The findings imply that GDM and the likelihood of depression during the postpartum phase are related. It was also found that there was a positive correlation between depression and the chance of having GDM. This emphasizes how the association between GDM and depression appears to be reciprocal. However, the association does not imply causation, and the data at hand do not allow for the establishment of causality. Subsequent studies ought to endeavor to show causative connections between GDM and depression as well as pinpoint shared underlying endocrine variables that may play a role in the genesis of both conditions. The available information that is now available is limited due to the complexity of the etiology of both GD and depression in pregnant women; nonetheless, prevention of both conditions depends on a better understanding of the link between GD and depression. The risk of bias in the included studies was moderate to high.

## Introduction and background

Over the past few decades, there has been an increase in the prevalence of gestational diabetes mellitus (GDM) [1]. GDM, which leaves moms and their offspring with numerous physical and mental difficulties, can be caused by a number of genetic, social, and psychological risk factors [2]. However, women are disproportionately more affected than men by depression, one of the most common psychiatric diseases [3]. Depression has become a global public health burden due to a notable rise in lifetime prevalence [4].

Depressive symptoms and syndromes that appear within the first year following childbirth are the hallmarks of postpartum depression (PPD) [5]. PPD is characterized by a hostile attitude toward infants, low self-esteem, exhaustion, melancholy, loss of appetite, self-blame, and humiliating feelings, along with other symptoms that endure for at least two weeks [6]. PPD can linger for up to six months following delivery [7] and often manifests four weeks after delivery [8]. According to earlier studies, PPD can persist for up to two years following birth [9]. Sadly, PPD has detrimental effects on women’s social and occupational functioning, and it can swiftly result in persistent or recurring chronic depression [10].

Additionally, PPD has an impact on the physical and mental health of the mother. Children of depressed moms show higher cortisol reactivity, less mature regulating behavior, lower social engagement, and more negative emotionality [11]. A strong correlation has been shown between PPD and a higher risk of maternal suicide [12]. Thus, identifying PPD’s prevalence and elucidating its risk factors is crucial for both treating and preventing PPD in society.

A prior meta-analysis of 11 longitudinal studies with 172,521 participants provided an overview of the association between diabetes and depression risk. According to the study, patients with type 2 diabetes had a considerable incident depression risk (pooled risk estimate = 1.24, 95% CI: 1.09, 1.40) [13]. The relationship between GDM and the likelihood of PPD or depressive symptoms has garnered more attention recently [14-16], although the results of earlier research were not entirely consistent. Therefore, by performing a systematic review, our goal was to examine the relationship between GDM and the likelihood of PPD symptoms.

The study aims to conduct a thorough review and evaluation of the body of research on the connection between GDM and the risk of developing symptoms of PPD. The study also aims to assess the potential risk factors associated with the co-occurrence of GDM and PPD and to examine the pathophysiological mechanisms underlying the association of GDM and PPD.

## Review

Methods

Study Design and Duration

The Preferred Reporting Items for Systematic reviews and Meta-Analyses (PRISMA) guidelines were followed in the conduct of this systematic review [17]. On February 1, 2024, the systematic review was started.

Search Strategy

An extensive search of the four primary databases - PubMed, Scopus, Web of Science, and Google Scholar - was done to find the relevant literature. We checked only English databases and took into account the unique requirements of each one. The subsequent keywords “postpartum depression,” “puerperal depression,” “postnatal depression,” “gestational diabetes mellitus,” and “pregnancy diabetes mellitus” were transformed into PubMed Mesh terms or subject terms in Scopus and used to locate the pertinent studies. The necessary keywords were matched by the Boolean operators “OR,” “AND,” and “NOT.” Among the search results were publications with full text in English and human studies.

Inclusion criteria: The study included women who were evaluated for PPD using either self-reported measures or validated diagnostic tools. Data on the prevalence of PPD and its associated risk factors were collected. The studies considered for inclusion were conducted between the years 2019 and 2024.

Exclusion criteria: Studies focusing on depression occurring within one week of delivery were excluded. Additionally, research involving diabetes mellitus other than GDM and publications in languages other than English, as well as animal studies, case reports, editorials, and commentaries, were not considered for this study.

Data Extraction

Rayyan (QCRI) was used to achieve duplicate identification and result verification for the search method [18]. The researchers added inclusion/exclusion criteria to the combined search results in order to evaluate the titles and abstracts’ relevancy. The reviewers gave each paper that met the inclusion requirements a thorough look. The authors talked about methods for resolving disputes. Using a previously created data extraction form, the authorized study was uploaded. The authors extracted data about the study titles, authors, study year, country, participants, follow-up duration, prevalence of PPD, risk factors, and main outcomes. A separate sheet was created for the risk of bias assessment.

Strategy for Data Synthesis

The qualitative assessment of the included studies was conducted by first extracting relevant data from each study, including study design, sample size, methodologies used, and key findings. This information was then synthesized and summarized in the form of summary tables to provide an overview of the different components and findings across studies.

After data collection, a thematic analysis approach was used to identify common themes and patterns across the included studies. This involved categorizing and organizing the extracted data into themes or categories based on similarities and differences in the study findings. This allowed for a deeper understanding of the overall patterns and trends within the research literature on the topic.

In addition, any discrepancies or conflicts in findings between studies were noted and discussed to understand the potential reasons for these variations. This process helped to provide a more comprehensive and nuanced interpretation of the research evidence and allowed for the identification of key areas of consensus or divergence within the literature.

Overall, the strategy for data synthesis involved a thorough examination and synthesis of the data collected from the included studies to provide a qualitative assessment of the components and research findings, ultimately leading to a comprehensive understanding of the current state of knowledge on the topic.

Risk of Bias Assessment

The Joanna Briggs Institute’s [19] key assessment criteria for studies providing prevalence data were applied in order to evaluate the research’s quality. This technique was used to evaluate studies using nine questions. The question was given a score of 1 if the answer was in the affirmative. Any response that was no, unclear, or not applicable received a score of 0. For overall quality, ratings of less than 4, 5 to 7, and more than 8 were regarded as low, moderate, and high quality, respectively. Scholars assessed the caliber of their research, and disagreements were settled by discussion.

Results

Search Results

**Figure 1:**
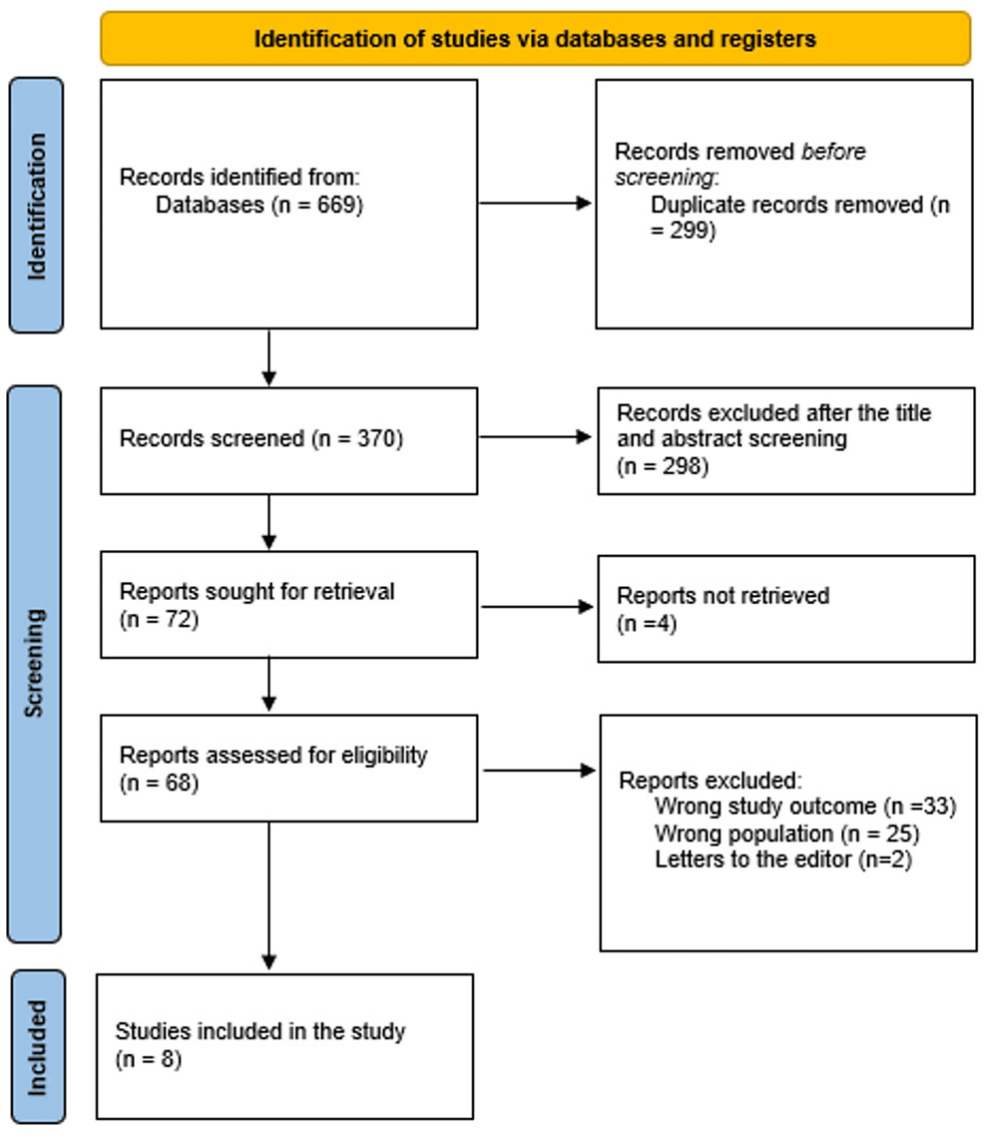
Study decision is summed up in a PRISMA diagram PRISMA, Preferred Reporting Items for Systematic reviews and Meta-Analyses

The systematic search yielded 669 study articles in total, of which 299 duplicates were eliminated. A total of 298 studies were eliminated after 370 studies had their titles and abstracts screened. Of the 72 reports that were requested to be retrieved, only four items were found. After screening 68 papers for full-text assessment, 33 were rejected due to incorrect study results, 25 were rejected due to incorrect population type, and two articles were editor’s letters. This systematic review had eight study papers that met the eligibility criteria. Figure 1 presents an overview of the process used to select the studies.

Characteristics of the Included Studies

The sociodemographic details of the research articles that are included are shown in Table 1. Our results included eight studies with a total of 4,209 women diagnosed with GDM and/or PPD [20-27]. Six studies were prospective in nature [20,21,23,24,26,27], one was a cross-sectional study [25], and one was case-control [22]. Two studies were conducted in the USA [22,24], one in India [20], one in Japan [21], one in the Netherlands [23], one in the UAE [25], one in China [26], and one in Belgium [27].

**Table 1:** Sociodemographic characteristics of the included participants GDM, gestational diabetes mellitus; NM, not mentioned

Study	Study design	Country	Participants (n)	Age (mean ± SD)
Singh et al. (2023) [20]	Prospective cohort	India	48	23.9 ± 3.7
Yamada et al. (2023) [21]	Prospective cohort	Japan	105	34.4 ± 4
Clark et al. (2019) [22]	Case-control	USA	382	18 to >43
Schmidt et al. (2019) [23]	Prospective cohort	The Netherlands	100	32.5 ± 4.1
Miller et al. (2021) [24]	Prospective cohort	USA	46	32
Alzarooni et al. (2024) [25]	Cross-sectional	UAE	186	18-45
Mak et al. (2019) [26]	Prospective cohort	China	1,499 (15.8% with GDM)	NM
Raets et al. (2021) [27]	Prospective cohort	Belgium	1,843 (12.5% with GDM)	31.4 ± 5.5

The clinical features are displayed in Table 2. Assessment of PPD was conducted using variable tools, including the Edinburgh Postnatal Depression Scale (EPDS) [20,21,25,26], the Patient Health Questionnaire-9 (PHQ-9) [24], and the Center for Epidemiologic Studies-Depression (CES-D) [27]. The prevalence of PPD in women diagnosed with GDM ranged from 6.5% [21] to 48.4% [22]. The included studies demonstrated that PPD was more likely to strike women with GDM [20-27]. One study reported that the most severe type of GDM is more likely to occur in those with a history of depression [22]. Perinatal depression during pregnancy can be strongly predicted by age, BMI, and a personal history of depression [22,25].

**Table 2:** Clinical characteristics and outcomes of the included studies CES-D, Center for Epidemiologic Studies-Depression; EPDS, Edinburgh Postnatal Depression Scale; GD, gestational diabetes; GDM, gestational diabetes mellitus; JBI, Joanna Briggs Institute; NM, not mentioned; OGTT, oral glucose tolerance test; PHQ-9, Patient Health Questionnaire-9; PPD, postpartum depression; SF-36, 36-Item Short Form Survey

Study	Follow-up duration (years)	Menopausal status, n (%)	Main outcomes	JBI
Singh et al. (2023) [20]	EPDS	7 (14.9%)	PPD was more likely to strike women with GDM. Therefore, it is possible to include the “at risk” strategy in standard antenatal treatment and evaluate all pregnant GDM women for PPD.	Moderate
Yamada et al. (2023) [21]	EPDS	6 (6.5%)	Pregnant women with GDM typically experience higher levels of anxiety and depression, as well as a greater mental load than pregnant women without GDM. PPD did not significantly vary in non-GDM women; however, it did decline quickly in GDM women following childbirth.	Moderate
Clark et al. (2019) [22]	NM	30 (48.4%)	The most severe type of GDM is more likely to occur in those with a history of depression; PPD is not significantly increased by GDM.	High
Schmidt et al. (2019) [23]	NM	12 (12%)	Women with GDM are probably going to have diabetic distress, and our research indicates that there may be a link between diabetes distress, parity, and unfavorable pregnancy outcomes in these women.	Moderate
Miller et al. (2021) [24]	PHQ-9	7 (15.2%)	Diagnoses of depression during the same pregnancy are strongly correlated with GDM. Given the harmful effects of depression on both mothers and fetuses, patients whose pregnancies are complicated by GDM may benefit from earlier referrals for counseling and improved screening for depression.	Moderate
Alzarooni et al. (2024) [25]	EPDS	64 (34.4%)	The occurrence of PPD among Emirati women with GDM suggests a statistically significant 50% increase in prognosis. The results of the study suggest that perinatal depression during pregnancy can be strongly predicted by age, BMI, and a personal history of depression.	Moderate
Mak et al. (2019) [26]	EPDS	NM	Women with GD had higher scores on the EPDS scale at one month (p = 0.02) and three months (p < 0.01) after delivery.	High
Raets et al. (2021) [27]	CES-D	NM	Depressed GDM women attended the postpartum OGTT less frequently (68.7% (33) vs. 87.6% (155), p = 0.002), stayed more often depressed (37.1% (13) vs. 12.4% (19), p < 0.001), and had poorer SF-36 scores after giving birth than women without depressive symptoms.	Moderate

Discussion

Depression has been linked to a higher risk of adverse pregnancy results and is a common perinatal problem [28,29]. This review found that PPD was more likely to strike women with GDM [20-27]. One study reported that the most severe type of GDM is more likely to occur in those with a history of depression [22]. A meta-analysis by Arafa and Dong reported that GDM risk may also be higher in women who have had depression in the past [30]. Few studies have looked into the effects of having both antenatal depression and GDM on pregnancy outcomes, despite the fact that both conditions have been extensively examined as independent risk factors for unfavorable perinatal outcomes. The association between the two disorders has to be better studied in order to potentially lessen perinatal problems in this high-risk population. Also, more information is required to determine how depression during pregnancy affects the quality of life after giving birth in both GDM-afflicted women.

Nevertheless, because of the inconsistent findings across the publications, the limited sample size of certain investigations, and the use of depressive symptoms rather than diagnostic methods to diagnose depression, a systematic review [31] was unable to come to an agreement regarding the association between GD and depression. It should be mentioned that this specific study, which gathered publications from 1995 to 2015, was among the first systematic reviews. Publications started to rise after 2015. Uncertainty surrounds the depression-related mechanisms that increase the risk of gestational diabetes in women. Immune dysfunction associated with depression biologically stimulates the sympathetic nervous system and the hypothalamic-pituitary-adrenal axis, increasing the release of stress hormones and inflammatory cytokines. These may cause insulin resistance by interacting with pancreatic β cells. Consequently, GD risk may rise as a result of consistent elevations in pro-inflammatory cytokines and adipokines linked to depression [30]. Furthermore, a number of lifestyle decisions, including inactivity and a sedentary way of living, are linked to depression and raise the risk of diabetes [30].

In the current study, the prevalence of PPD in women diagnosed with GDM ranged from 6.5% [21] to 48.4% [22]. The wide range of reported prevalence rates of prenatal depression is likely caused by variations in the population under study, the time frame employed to measure prevalence, and the diagnosis of depression. We also found that perinatal depression during pregnancy can be strongly predicted by age, BMI, and a personal history of depression [22,25].

To completely comprehend the reciprocal association between GD and depression, more research is required. Yet, the levels of many hormones rise during pregnancy, including those related to reproduction, such as progesterone and estradiol, as well as those from other biological systems, such as cortisol, thyroid-stimulating hormone, corticotropin-releasing hormone, or prolactin; these hormones then return to normal levels following parturition [31]. These abrupt fluctuations in hormones are offset by balancing interventions and may have implications for mental health and endocrine disorders like diabetes [32]. Studies have discovered a bidirectional relationship between depression and endocrine disorders [30]. However, these disorders might also be caused by other sources. Future research that makes use of psychiatric problems and the history of endocrine diseases will be required to further clarify the processes behind the relationship between diabetes and depression. However, these disorders might also be caused by other sources. Future research that uses women’s medical histories and psychological states before, during, and after pregnancy will be required to better understand the mechanisms behind the relationship between diabetes and depression. The disparity in the research included in this study was one of its weaknesses.

There are various advantages to this study. We offer comprehensive data on clinical and biochemical features, pregnancy outcomes, and postpartum quality of life from a sizable prospective cohort. Quality of life and depression symptoms were evaluated using validated questionnaires. One major limitation is the potential bias present in the included studies, as they varied in study design, population demographics, and measurement methods. Additionally, the variability in diagnostic criteria for gestational diabetes and PPD across studies may have impacted the consistency of the findings. The cross-sectional assessment of depressive symptoms in the study is one of its limitations, as it prevents the investigation of a long-term association between depression and GDM. The assessment was conducted using variable tools, including the EPDS [20,21,25,26], PHQ-9 [24], and CES-D [27], which may be a source of bias. Furthermore, the inadequate control of potential confounding factors that could influence the relationship between gestational diabetes and PPD is another important limitation to acknowledge.

There are several important implications that should be considered for future research in this area. It is essential to conduct more well-designed prospective cohort studies to provide a deeper understanding of the relationship between gestational diabetes and PPD. Standardizing diagnostic criteria for gestational diabetes and PPD would be beneficial in allowing for more accurate comparisons across studies. Exploring potential biological mechanisms linking gestational diabetes and PPD, such as hormonal changes and inflammation, could provide valuable insights into the underlying mechanisms at play. Developing targeted interventions for women with gestational diabetes to prevent or mitigate PPD should be a priority for future research and clinical practice. Lastly, investigating the long-term implications of gestational diabetes on maternal mental health beyond the postpartum period would be valuable in understanding the full impact of gestational diabetes on women’s mental well-being.

## Conclusions

The findings imply that GDM and the likelihood of depression during the postpartum phase are related. It was also found that there was a positive correlation between depression and the chance of having GDM. This emphasizes how the association between GDM and depression appears to be reciprocal. However, the association does not imply causation, and the data at hand do not allow for the establishment of causality. Subsequent studies ought to endeavor to show causative connections between GDM and depression as well as pinpoint shared underlying endocrine variables that may play a role in the genesis of both conditions. The information that is now available is limited due to the complexity of the etiology of both GD and depression in pregnant women; nonetheless, prevention of both conditions depends on a better understanding of the link between GD and depression.
